# Case of Suffocation Produced by Sulphuric Acid

**Published:** 1853-05

**Authors:** 


*Case of Suffocation Produced by Sulphuric Acid.*—The interest of this case lies in the fact that death was caused by the action of the acid on the respiratory passages, without touching the organs of digestion.

A woman was found dead in her bed-room; she was seated on a chair, her head bent back, resting on the bed, and drooping towards her right shoulder. Her mouth was full of tough mucus, the lips, teeth, and gums presented the appearance of being corroded by an acid. Her right hand, placed across the body, held a small phial labelled *sulphuric acid, (poison.)* It contained about half a drachm of the acid. Death had occurred a considerable time before; the extremities were already cold.

*Autopsy.*—The tongue was contracted, the epiglottis corroded and reduced to a little triangular tongue with indented edges. The vocal cords were destroyed, particularly the right. The mucous membrane of the trachea was also removed, and the cartilages had the appearance of being dissected. The acid had penetrated into the two lungs, attacked the parenchyma, perforated the left pleura, destroyed the costal layer, and roughened the subjacent ribs.

On the surface of the lung there was a layer of sulphate of lime; the arch of the aorta, the superior cava, and the innominata veins contained clots having the appearance of dried wax. No trace of acid could be found in the stomach or œsophagus. The epithelial lining was quite entire, and the stomach only contained some mucus.

Dr. Gall remarks, "that he believes no similar case has been recorded. It is well known that the impression produced instantaneously on the tongue by the caustic, occasionally causes contraction of the pharynx sufficient to prevent deglutition, and death has been caused by the progress of the inflammation and tumefaction of the pharyngeal mucous lining and tonsils, when not a drop of acid has entered the stomach; but no case has hitherto been recorded in which the acid had penetrated into the bronchial tubes. Could this have been caused by a fit of coughing raising the epiglottis, and drawing the liquid into the trachea? Probably enough, and the acid afterwards would have run gradually into the minute ramifications of the tubes, causing corrosion as it passed along."—*Glasgow Med. Journ. from Dr. Gall in Journal de Chemie Médicale.*

